# A survey of parental experiences and perceptions of NAVA in neonatal intensive care

**DOI:** 10.1007/s00431-025-06718-0

**Published:** 2026-01-09

**Authors:** Donna Tolentino, Laura De-Rooy, Anay Kulkarni, Sandeep Shetty

**Affiliations:** 1 Neonatal Intensive Care Centre, St George’s University Hospitals NHS Foundation Trust, London, UK; 2 George’s University of London, London, UK; 3 Department of Women and Children’s Health, School of Life Course Sciences, Faculty of Life Sciences and Medicine, King’s College London, London, UK

**Keywords:** Neurally adjusted ventilatory assist, NAVA, NIV-NAVA, Neonatal ventilation, Parental perceptions, Patient–ventilator synchrony, Family-centred care

## Abstract

To evaluate parental perceptions and experiences of neurally adjusted ventilatory assist (NAVA) in neonatal intensive care, focusing on understanding, comfort, and willingness to recommend its use. A survey of parents whose infants received NAVA or Non-invasive ventilation (NIV) NAVA at a tertiary NICU between January 2024 and July 2025. St George’s University Hospitals NHS Foundation Trust, London, UK. Fifty parents were invited; 32 returned completed surveys (response rate: 64%). Parent-reported understanding of ventilation modes, perceptions of infant comfort and willingness to recommend NAVA. Most respondents (81%, n = 26) felt staff explained different modes of breathing support clearly; 4 (13%) found explanations unclear, and 2 (6%) received none. Seventeen (53%) parents felt their baby was calmer and more settled during NAVA/NIV NAVA compared with other modes; 11 (34%) noticed no difference, and 4 (13%) perceived less comfort. The NAVA catheter scored a mean of 3.77/5 for comfort. Twenty-three (78%) stated they were “very likely” to recommend NAVA, 5 (16%) were “likely,” 1 (6%) was “neutral,” and 2 (12%) would not recommend it.

*Conclusion*: Most parents reported positive perceptions of NAVA, with improved comfort compared with conventional modes.

**Supplementary Information:**

The online version contains supplementary material available at 10.1007/s00431-025-06718-0.

## Introduction

Mechanical ventilation (MV), although lifesaving, is associated with chronic respiratory morbidity in both preterm and term born infants. New ventilation modes have been developed with the aim of minimising lung injury; these include invasive and non-invasive respiratory support strategies [1]. These can range from conventional invasive ventilation modes, such as pressure-regulated volume control (PRVC) and pressure control (PC) ventilation, to non-invasive methods, such as Heated, humidified, high-flow nasal cannula (HHFNC), continuous positive airway pressure (CPAP), non-invasive pressure control ventilation (NIV-PC) [1]. More recently, Neurally Adjusted Ventilatory Assist (NAVA) and its non-invasive variant (NIV-NAVA) have been introduced, offering a more synchronised form of support that uses the infant’s own diaphragmatic electrical activity to trigger and modulate breaths [2].

Studies comparing NAVA with conventional mechanical ventilation modes demonstrated that NAVA improved patient-ventilator interaction and comfort [3] and decreased peak inspiratory pressure (PIP) and mean airway pressure (MAP), work of breathing, oxygen requirement (FiO_2_) [4], sedation requirement [5] and episodes of apnoea [3]. Moreover, a review of ten studies comparing NAVA or NIV-NAVA to conventional respiratory support modes concluded that the application of NAVA appears to be safe and feasible in premature infants as no adverse events were reported [6]. In a randomised crossover study comparing NAVA with conventional ventilation modes in infants with evolving or established Bronchopulmonary Dysplasia (BPD), NAVA improved oxygenation by reducing oxygenation index (OI), FiO_2_, peak inspiratory pressure (PIP) and mean airway pressure (MAP) and compliance was higher [7]. In a retrospective case control study, infants with evolving BPD on NAVA/NIV NAVA had lower extubation failure rates (*p* = 0.002), shorter durations of invasive ventilation (*p* = 0.046), total duration of invasive and non-invasive ventilation (*p* = 0.026) and total length of hospital stay (*p* = 0.019). There were no significant differences, however, in the rates of BPD or home oxygen [7]. Data suggest that NAVA/NIV and NAVA ventilation in preterm infants improves their growth trajectory at the time of discharge, probably due to the improved synchronisation and patient comfort [8]. Evidence suggests that NAVA may reduce PIP, improve patient–ventilator synchrony, decrease the work of breathing, and promote greater comfort compared with conventional modes [7].

Effective communication between NICU staff and parents plays a central role in supporting families during their infant’s hospital journey [9]. The family-integrated care (FICare) model offers a framework to actively involve parents in the care of their baby during their neonatal unit admission and to encourage collaboration between parents and healthcare professionals [9]. In the context of advanced ventilation modes such as NAVA, providing clear information on how the mode works, and how this support might affect infant comfort and bonding is particularly important.

This survey was developed to capture the experiences of parents whose infants received NAVA or NIV-NAVA. By gathering these insights, this study aims to identify opportunities to improve communication, education, and support for families, while highlighting parental experience with advanced ventilatory care in neonates.

## Methods

NAVA was introduced at St George’s Hospital in June 2019. Since then, a total of 200 patients received NAVA/NIV NAVA. For this survey, we targeted parents of infants who had been offered NAVA between January 2024 and July 2025, to minimise recall bias. Parents of infants who had died were not contacted, in line with ethical considerations. Eligibility was confirmed by cross-referencing records with the neonatal database (BadgerNet).

An online questionnaire (Appendix S1) designed in collaboration with a neonatal psychologist, accessible via a QR code linking to Google Forms, was distributed by post to 50 eligible parents. The survey explored parents’ recollections of the different respiratory support modes used, their understanding of how these methods help their baby, and their perceptions of their baby’s comfort and interaction during NAVA compared with other modes. It also examined views on the NAVA-specific feeding tube and willingness to recommend NAVA to others. Demographic details were also collected.

Data obtained were analysed descriptively, with results presented as percentages. Where appropriate, bar plots were used to visualise the distribution of responses.

This project was registered as an audit with St George’s University Hospitals NHS Foundation Trust Audit Department (Registration number AUDI004527).

## Results

**Fig. 1:**
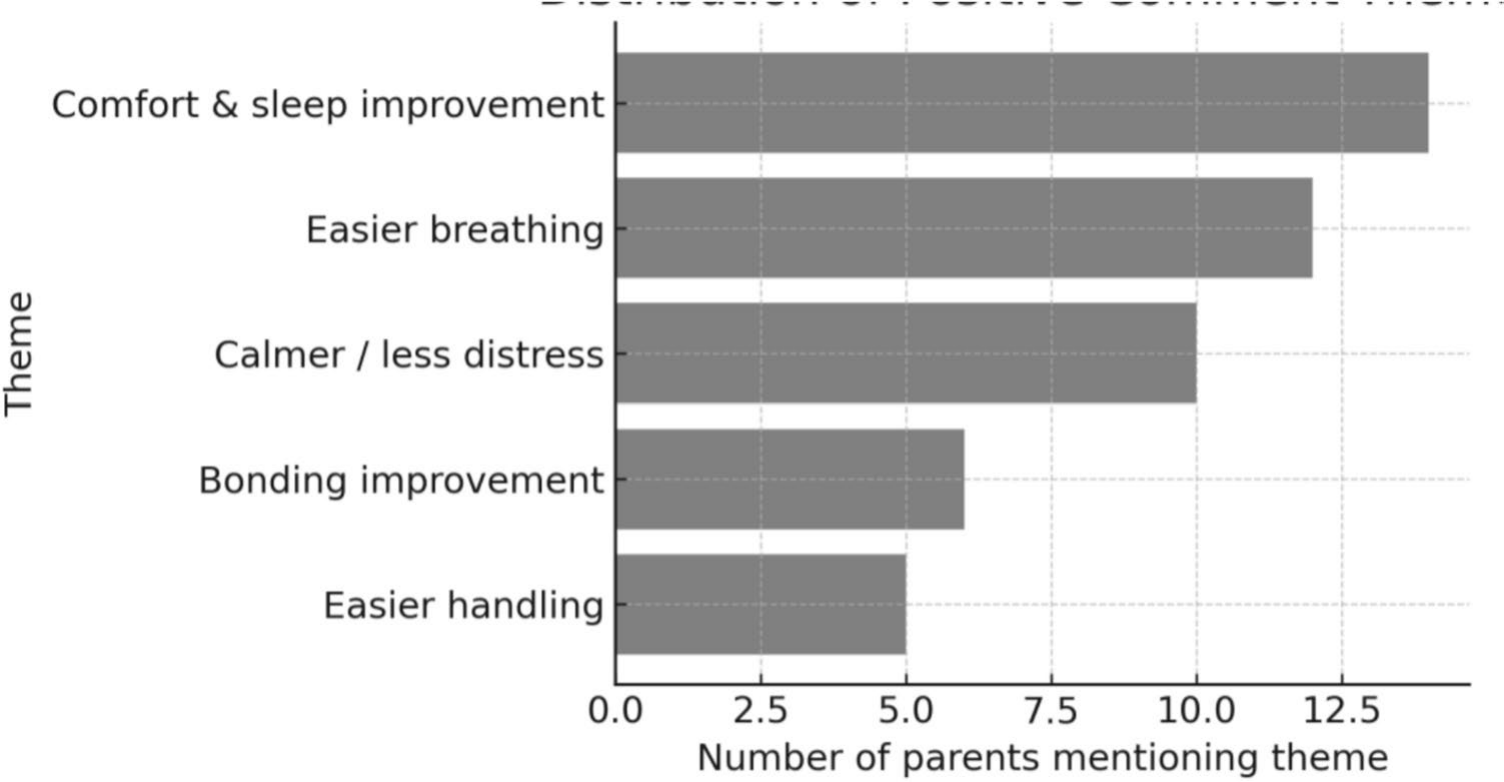
Distribution of Free-text Responses Themes

A total of 50 parents were invited to participate; 32 completed surveys were returned (response rate: 64%) (Table 1). Parents reported their infants had received various forms of respiratory support during admission, including NAVA (n = 24, 75%), NIV NAVA (n = 26, 81%), PRVC (n = 23, 72%), CPAP (n = 22, 69%), pressure control (PC) ventilation (n = 19, 59%), and NIV PC (n = 23, 72%); 3 (9%) parents reported other modes. Most respondents (81%, n = 26) felt staff explained different modes of breathing support clearly; 4 (13%) found explanations unclear, and 2 (6%) received none. Seventeen (53%) parents felt their baby was calmer and more settled during NAVA/NIV NAVA compared with other modes; 11 (34%) noticed no difference, and 4 (13%) perceived less comfort. The NAVA catheter scored a mean of 3.77/5 for comfort. Positive observations included *“longer, deeper sleep,” “less fighting the machine,”* and *“breathing seemed easier”* (Fig. 1). Twenty-three parents (78%) stated they were “very likely” to recommend NAVA, 5 (16%) were “likely,” 1 (6%) was “neutral,” and 2 (12%) would not recommend it. Respondents included mothers (n = 19, 59%), fathers (n = 3, 9%), and both parents together (n = 10, 31%). Ethnic backgrounds included Black Caribbean, Black British, and White/Other.

**Table 1:** Parent survey responses (n = 32)

Outcome category	Subcategory	n (%)
Breathing support modes	NAVA	24 (75)
	NIV NAVA	26 (81)
	PRVC	23 (72)
	CPAP	22 (69)
	Pressure support ventilation	19 (59)
	NIV PC	23 (72)
	Other modes	3 (9)
Staff explanations	Clear/good understanding	26 (81)
	Unclear	4 (13)
	None	2 (6)
Comfort (NAVA/NIV NAVA)	More comfortable	17 (53)
	Less comfortable	4 (13)
	No difference	11 (34)
NAVA catheter comfort	Mean (SD) score (1–5)	3.77
Recommendation	Very likely	23 (72)
	Likely	5 (16)
	Neutral	1 (3)
	Unlikely	2 (6)

## Discussion

This single-centre survey found that parents whose infants received NAVA or NIV-NAVA generally perceived these modes as providing improved comfort compared with conventional ventilation. More than half of respondents reported their infant was calmer on NAVA-based support. Qualitative comments suggested perceived benefits of NAVA included longer sleep periods, reduced visible effort of breathing, and greater overall calmness. While subjective, such perceptions are aligned with clinician observations of reduced respiratory drive variability and more stable breathing patterns in infants on NAVA [10]. Continuous electrical activity of the diaphragm (Edi) catheter readings and ventilatory parameters recorded during 65 skin-to-skin contact (SSC), peak Edi (µV) was significantly reduced at end SSC (median 11.5 [2.7–38.7] vs. 15.8 [4.0–36.6], *p* < 0.001), as was mean airway pressure (*P*mean, cmH₂O) (9.7 [7.3–15.4] vs. 10.3 [7.5–15.5], *p* = 0.008), and respiratory rate (breaths/min) (52.9 [31.1–78.1] vs. 53.4 [35.1–74.1], *p* = 0.031) [11].

The majority of respondents felt that staff explained breathing support modes clearly, underscoring the role of effective communication in supporting family-centred care [12].

The high proportion (72%) who were “very likely” to recommend NAVA to others indicates overall satisfaction, and this is consistent with wider evidence linking perceived infant comfort to parental satisfaction [11, 13]. Family experience is increasingly recognised as an important quality metric in neonatal care [14]. Although our survey was not designed to assess clinical outcomes, positive parental perceptions may contribute to enhanced parent–infant bonding and participation in care, which are themselves associated with improved developmental and psychosocial outcomes [15].

This study has limitations. The sample size was modest and limited to a single centre, and participation was restricted to parents whose infants survived to discharge. Recall bias may have influenced responses, though the survey was timed to minimise long-term recall error. Despite these limitations, the findings highlight the value of integrating parental perspectives into evaluations of respiratory support modalities. We reported quantitative and qualitative data, which gave a more comprehensive understanding of visiting policies.

In conclusion, parents perceived NAVA and NIV-NAVA as providing greater comfort and valued clear staff communication. As NICU care moves towards more personalised and family-centred approaches, incorporating parental perspectives into assessments of new technologies will be essential for ensuring that innovation meets both clinical and family needs.

## Supplementary Information

Below is the link to the electronic supplementary material.

Supplementary Material 1 (PDF 153 KB)

## References

[1] Kaltsogianni O, Dassios T, Greenough A (2023) Neonatal respiratory support strategies-short and long-term respiratory outcomes. Front Pediatr 11:1212074 37565243 10.3389/fped.2023.1212074 PMC10410156

[2] Stein H, Firestone K (2014) Application of neurally adjusted ventilatory assist in neonates. Semin Fetal Neonatal Med 19(1):60–69 24238745 10.1016/j.siny.2013.09.005

[3] Mally PV, Beck J, Sinderby C, Caprio M, Bailey SM (2018) Neural breathing pattern and patient-ventilator interaction during neurally adjusted ventilatory assist and conventional ventilation in newborns. Pediatr Crit Care Med 19(1):48–55 29189671 10.1097/PCC.0000000000001385

[4] Jung YH, Kim HS, Lee J, Shin SH, Kim EK, Choi JH (2016) Neurally adjusted ventilatory assist in preterm infants with established or evolving bronchopulmonary dysplasia on high-intensity mechanical ventilatory support: a single-center experience. Pediatr Crit Care Med 17(12):1142–1146 27918385 10.1097/PCC.0000000000000981

[5] Rong X, Liang F, Li YJ et al (2020) Application of Neurally Adjusted Ventilatory Assist in Premature Neonates Less Than 1,500 Grams With Established or Evolving Bronchopulmonary Dysplasia. Front Pediatr 8:110 32266188 10.3389/fped.2020.00110 PMC7105827

[6] Fang SJ, Chen CC, Liao DL, Chung MY (2023) Neurally adjusted ventilatory assist in infants: a review article. Pediatr Neonatol 64(1):5–11 36272922 10.1016/j.pedneo.2022.09.003

[7] Shetty S, Evans K, Cornuaud P, Kulkarni A, Duffy D, Greenough A (2021) Neurally adjusted ventilatory assist in very prematurely born infants with evolving/established bronchopulmonary dysplasia. AJP Rep 11(4):e127–e131 34849284 10.1055/s-0041-1739458 PMC8608553

[8] Benn K, De Rooy L, Cornuaud P, Kulkarni A, Shetty S (2022) Improved nutritional outcomes with neurally adjusted ventilatory assist (NAVA) in premature infants: a single tertiary neonatal unit’s experience. Eur J Pediatr 181(5):2155–2159 35194652 10.1007/s00431-022-04411-0 PMC9056442

[9] Waddington C, van Veenendaal NR, O’Brien K, Patel N (2021) International Steering Committee for Family Integrated C. Family integrated care: Supporting parents as primary caregivers in the neonatal intensive care unit. Pediatr Investig 5(2):148–154 34179713 10.1002/ped4.12277 PMC8212757

[10] Breatnach C, Conlon NP, Stack M, Healy M, O’Hare BP (2010) A prospective crossover comparison of neurally adjusted ventilatory assist and pressure-support ventilation in a pediatric and neonatal intensive care unit population. Pediatr Crit Care Med 11(1):7–11 19593246 10.1097/PCC.0b013e3181b0630f

[11] Serrano-Llop A, De-Rooy L, Duffy D, Kulkarni A, Shetty S (2023) Improved respiratory parameters with skin-to-skin contact in premature infants with bronchopulmonary dysplasia on NIV-NAVA. Acta Paediatr 112(4):647–651 36541864 10.1111/apa.16638

[12] Gooding JS, Cooper LG, Blaine AI, Franck LS, Howse JL, Berns SD (2011) Family support and family-centered care in the neonatal intensive care unit: origins, advances, impact. Semin Perinatol 35(1):20–28 21255703 10.1053/j.semperi.2010.10.004

[13] Singh AK, Das KS, Ranjan A, Sahu SS, Lakra PS, Kumar A (2024) Assessing the Impact of Different Levels of Parental Involvement in the NICU on Neonatal Outcomes and Parental Mental Health. J Pharm Bioallied Sci 16(Suppl 3):S2836–S2838 39346450 10.4103/jpbs.jpbs_350_24 PMC11426676

[14] van Wyk L, Majiza AP, Ely CSE, Singer LT (2024) Psychological distress in the neonatal intensive care unit: a meta-review. Pediatr Res 96(6):1510–1518 39327462 10.1038/s41390-024-03599-1 PMC11624136

[15] Soghier LM, Kritikos KI, Carty CL et al (2020) Parental Depression Symptoms at Neonatal Intensive Care Unit Discharge and Associated Risk Factors. J Pediatr 227(163–169):e161 10.1016/j.jpeds.2020.07.040 32681990

