# Supplementary material for: A survey of parental experiences and perceptions of NAVA in neonatal intensive care
Source: Eur J Pediatr. 2026 Jan 9;185(1):65. doi: 10.1007/s00431-025-06718-0 (PMC12789205; doi:10.1007/s00431-025-06718-0)
Supplement: Supplementary file 1 — Supplementary Material 1 (PDF 153 KB) [file 431_2025_6718_MOESM1_ESM.pdf]

Dear Parents and Carers,

Thank you for taking the time to read this information about NAVA breathing support and filling in our short questionnaire. St George's neonatal unit has pioneered the use of this new technology in the UK in supporting the tiniest preterm babies and we want to find out what parents think about their baby's experience. This will help us to understand more about the impact of this emerging technology and guide us in improving staff and parent education. It should take about 5 minutes to fill in.

Background information:

NAVA stands for Neurally Adjusted Ventilatory Assist. It is a relatively newer mode of breathing support, which works by picking up electric signals from the baby's diaphragm (the breathing muscle) as the baby breathes. The machine can adjust how much support it gives the baby depending on the strength of the signal and the baby's own effort. This means baby and machine work better together and the baby uses less energy to breath and has more energy to grow and develop. We can use NAVA both when the baby is breathing through a tube placed in the windpipe (intubated) or when the baby is getting support via a face mask or nasal prongs (non-invasively ventilated).

Thank you so much for completing this questionnaire. Some of these questions may have raised difficult memories and feelings of your time on the unit. If you would like to talk to our psychology team about finding local wellbeing support, please email or contact your GP.'

If you have any further questions or comments about this questionnaire, please contact us on:

When you submit this form, it will not automatically collect your details like name and email address unless you provide it yourself.

1. Your baby may have received different methods of breathing support while on the neonatal unit. Please tick all the methods of breathing support that you remember your baby received

- o PRVC- type of **invasive breathing support** (through a breathing tube in the mouth) in which the tidal volume (amount of air your baby inhales in a normal breath) are set on the ventilator and the pressure delivered by the ventilator varies, to achieve the set tidal volume.
- o Pressure Support- mode of **invasive breathing support** whereby the airway pressure that is delivered by the ventilator is set by a member of the medical team. The tidal volume may vary to achieve the set pressure.
- o NAVA- mode of **invasive breathing support** that works by picking up electrical signals from baby's diaphragm. It requires a special feeding tube called a "NAVA catheter".
- o CPAP- is a type of **non-invasive breathing support** (through nose via mask/prongs). The pressure is set on the machine to deliver a continuous pressure during both inspiration and expiration.

- NIV NAVA- type of **non-invasive breathing suppor**t (via nose with mask or prongs) in which the medical team sets the pressures to be delivered to the baby when the baby breathes in.
- NIV PC- type of **non-invasive breathing support** (via nose with a mask or prongs) that supports baby's breathing using the electrical signal from baby's diaphragm. It needs special feeding tube called a "NAVA catheter".

2. **Did a doctor or nurse explain to you** how these different methods of breathing support help your baby? Please tick which best reflects how you feel?

- Yes, staff explained the different methods of breathing support to me and I feel I had a good understanding.
- Staff did explained the different methods of breathing support to me but it was unclear and I did not always understand.
- No, staff did not explain the different methods of breathing support to me
- I don't remember staff explaining different methods of breathing support.

3. When your baby was on NAVA or NIV NAVA support compared to other support, was there a change in their responses to touch, cuddles, and interaction with you? Please tick which of these statements best describes your experience

- My baby appeared calmer and more settled when on NAVA or NIV NAVA support as compared to any other method of breathing support.
- My baby appeared less comfortable when on NAVA or NIV NAVA support as compared to any other method of breathing support.
- I did not notice any difference in my baby's state during NAVA or NIV NAVA support as compared to any other method of breathing support.

4. To deliver NAVA, staff place a special feeding tube through the nose or mouth into the stomach. This feeding tube works both as a feeding tube and has small sensors to detect diaphragmatic movement to trigger the breathing machine.

   On a scale of 1-5 please let us know how you felt the feeding tube with sensor affected your baby as compared to your baby's standard feeding tube.

   1 star: More uncomfortable
   2 stars: Less Uncomfortable
   3 stars: No Change
   4 stars: Comfortable
   5 stars: Comfortable and settled

5. If you had a friend or family member whose baby might receive NAVA, would you recommend it?
   - Very likely
   - Likely
   - Neutral
   - Would not recommend
   - Would strongly not recommend

6. Please provide your email address below if you would like to be involved in future research

7. The next questions will provide the researcher basic information about who took part in the survey. Are you male or female?

8. What is your ethnic background?

9. Who is filling out this survey?
   - Mother
   - Father
   - Both parents
